# Disease-Associated SNPs in Inflammation-Related lncRNAs

**DOI:** 10.3389/fimmu.2019.00420

**Published:** 2019-03-08

**Authors:** Ainara Castellanos-Rubio, Sankar Ghosh

**Affiliations:** ^1^Immunogenetics Research Laboratory, Department of Genetics, Physical Anthropology and Animal Physiology, University of the Basque Country, UPV/EHU, Leioa, Spain; ^2^Functional Studies in Celiac Disease Group, BioCruces Health Research Institute, Barakaldo, Spain; ^3^Department of Microbiology and Immunology, Vagelos College of Physicians and Surgeons, Columbia University, New York, NY, United States

**Keywords:** lncRNA, linc RNA, inflammation, inflammatory disease, GWAS, SNP

## Abstract

Immune-mediated diseases, such as celiac disease, type 1 diabetes or multiple sclerosis, are a clinically heterogeneous group of diseases that share many key genetic triggers. Although the pathogenic mechanisms responsible for the development of immune mediated disorders is not totally understood, high-throughput genomic studies, such as GWAS and Immunochip, performed in the past few years have provided intriguing hints about underlying mechanisms and pathways that lead to disease. More than a hundred gene variants associated with disease susceptibility have been identified through such studies, but the progress toward understanding the underlying mechanisms has been slow. The majority of the identified risk variants are located in non-coding regions of the genome making it difficult to assign a molecular function to the SNPs. However, recent studies have revealed that many of the non-coding regions bearing disease-associated SNPs generate long non-coding RNAs (lncRNAs). LncRNAs have been implicated in several inflammatory diseases, and many of them have been shown to function as regulators of gene expression. Many of the disease associated SNPs located in lncRNAs modify their secondary structure, or influence expression levels, thereby affecting their regulatory function, hence contributing to the development of disease.

## Introduction

Immune mediated disorders, such as celiac disease (CeD), inflammatory bowel diseases (IBD), atherosclerosis, rheumatoid arthritis (RA), type 1 diabetes (T1D) or multiple sclerosis (MS) among others, are a group of clinically heterogeneous diseases caused by dysfunction of the immune system. These disorders, share underlying pathogenic mechanisms that are not totally understood, although the general belief is that they develop due to an imbalance in the interaction between genetic and environmental factors (1, 2).

Immune mediated disorders share dysregulation of many key regulatory pathways and techniques, such as genome wide association studies (GWAS) coupled with next generation sequencing (NGS), have significantly increased our knowledge of genetic factors underlying such diseases (3). In the past decade or so, hundreds of risk alleles, both common and disease specific, have been identified by GWAS. Moreover, using the Immunochip platform in which 200,000 polymorphisms in 186 immune disease related regions were analyzed, additional immune disease associated variants were identified that revealed common susceptibility loci for several of these diseases (4–7). While these studies have helped identify immune disease conferring gene variants, the progress toward the understanding of the underlying mechanisms has remained limited. This difficulty is particularly exacerbated by the fact that around 90% of the SNPs associated with these diseases are located in non-coding regions, making it difficult to link them to specific biological pathways (8–10).

Advances in the sequencing and annotation of the human genome have revealed that many non-coding regions of the genome encode long non-coding RNAs (lncRNAs). The importance of lncRNA molecules in different biological processes is beginning to be appreciated although there is much that remains to be understood. LncRNAs are RNA molecules longer than 200 bp in length with no protein-coding potential. LncRNA expression is generally cell-lineage specific and they have diverse and still not very well-characterized mechanisms of action. The emerging view is that lncRNAs are fundamental regulators of transcription as they have been shown to control every level of the gene expression program. LncRNAs have been shown to control processes like protein synthesis, RNA maturation, and transport to regulate genes post-transcriptionally and they are also involved in transcriptional gene silencing through regulating the chromatin structure (11–13).

Many lncRNAs are enriched for disease-associated SNPs, suggesting that these SNPs might alter the function of lncRNAs e.g., by altering their secondary structures (14). Moreover, expression profile analyses of lncRNAs located in autoimmune disease-associated regions showed that lncRNAs are enriched in these loci, suggesting that lncRNAs may be crucial for interpreting GWAS findings (15). Disease associated SNPs can modify the lncRNA sequence or alter their gene expression levels, affecting their regulatory capacity, and alterations in the structure and function of lncRNAs have been associated with several immune-mediated diseases. However, the precise mechanism by which lncRNA variants contribute to the pathogenesis of disease remains unknown in the majority of cases (16).

**Figure 1:**
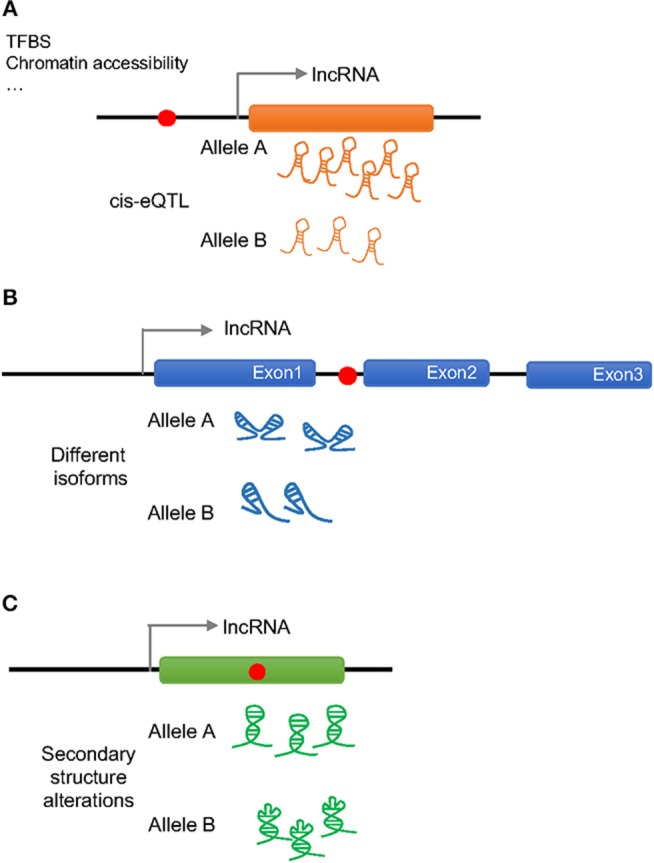
Possible effects of a disease associated SNP on lncRNA regulation and function. Red dots represent disease associated SNPs. **(A)** A SNP in the promoter region of a lncRNA can change the amount of transcribed lncRNA (cis-eQTL) by altering the binding of transcription factor binding sites (TFBS) or the chromatin accessibility altering downstream effects. **(B)** An intronic SNP in a lncRNA can influence the splicing of the lncRNA generating different isoforms that will act differently. **(C)** A SNP in the exonic sequence of a lncRNA can change its secondary structure altering the binding to the molecular partners.

As previously done with protein coding genes, intergenic SNPs have been analyzed in the context of lncRNA expression quantitative trait loci (eQTLs), namely, genetic variants that would explain variation in the lncRNA expression levels (17). More than 100 cis-eQTLs have been found in different tissues that appear to regulate the expression of nearby lncRNAs. In general, these eQTLs are lncRNA specific and do not regulate the expression of neighboring protein coding genes, but since lncRNAs can regulate the expression of protein-coding genes, both, located close by or farther away in the genome, it is possible that these SNPs also influence the function of protein-coding genes. Moreover, a considerable number of the lncRNA cis-eQTLs belong to disease-associated SNPs, making lncRNAs a potential link between non-coding SNPs and the expression of protein-coding genes (18–21) (Figure 1A).

Disease associated SNPs have been also suggested to be involved in “splicing models,” in which the presence of different alleles would influence the splicing of the lncRNA by regulating exon skipping (22) (Figure 1B). In this way, different isoforms of the lncRNA would present different affinity for their binding partners affecting the regulation of downstream events. It has been observed, that when human endothelial cells are stimulated with lipopolysaccharide (LPS) several lncRNAs show splice variant-specific expression at different stimulation time points (23), underlining the importance of SNP regulated splicing in lncRNA function.

As it is generally believed that lncRNA molecules adopt specific secondary and tertiary structures to execute their functions, it is likely that disease associated SNPs have an impact on lncRNA structure (Figure 1C). The analysis of secondary structures has largely been performed using computational tools, and several tools can predict changes in lncRNA structures caused by the presence of different alleles of a certain SNP (24–26). For example, GWAS SNPs associated with IBD and T1D have been shown to disrupt the structure of an lncRNA implicated in the pathogenesis of both diseases, which associates with the *BACH2* gene (27). However, this field is still in its infancy and the principles that underlie the impact of SNPs on lncRNA structure and function remains to be fully established.

In this article, we have reviewed the link between four intergenic GWAS variants that are located within lncRNA sequences, which have been associated with inflammatory diseases, and we discuss the studies that have been carried out to characterize their contribution to the development of disease pathogenesis. As of now, these four inflammatory-disease associated SNPs are the best mechanistically characterized in the context of lncRNA function.

## *Lnc13* and Celiac Disease Susceptibility

Celiac disease is a complex, chronic, immune-mediated disease that affects ~1% of the population and develops in genetically susceptible individuals in response to ingested gluten proteins from wheat, barley, and rye (28). The strongest genetic association, around 40% of the genetic risk (29), maps to the human leukocyte antigen (HLA) region in chromosome 6p21, and virtually all CeD patients carry HLA-DQ2 or HLA-DQ8 heterodimers (30, 31).

Two GWA studies, together with the Immunochip project, have identified a total of 39 non-HLA loci associated with the genetic risk of CeD (32–34). Only 3 of the CeD associated SNPs are linked to protein-altering variants located in exonic regions, although some potentially causative coding genes have been proposed, mainly related to the immune response, due to the existence of signals near their 5′ or 3′ regulatory regions. Although some lncRNAs have been related to celiac disease pathogenesis due to the location of an associated SNP within their transcriptional region, and differential expression found in samples from CeD patients (35, 36), the exact mechanism by which they contribute to disease development is not understood.

The only functionally characterized lncRNA harboring a CeD associated intergenic SNP so far has been found linked to the NF-κB pathway (37), which is known to be constitutively active in the CeD mucosa (38, 39). This lncRNA, named *lnc13*, harbors the SNP rs917997 and it is located in the associated region 2q12, with the sense sequence overlapping the coding gene *IL18RAP* that had been proposed, but never firmly confirmed, as the functional candidate gene in the region (40–42).

**Figure 2:**
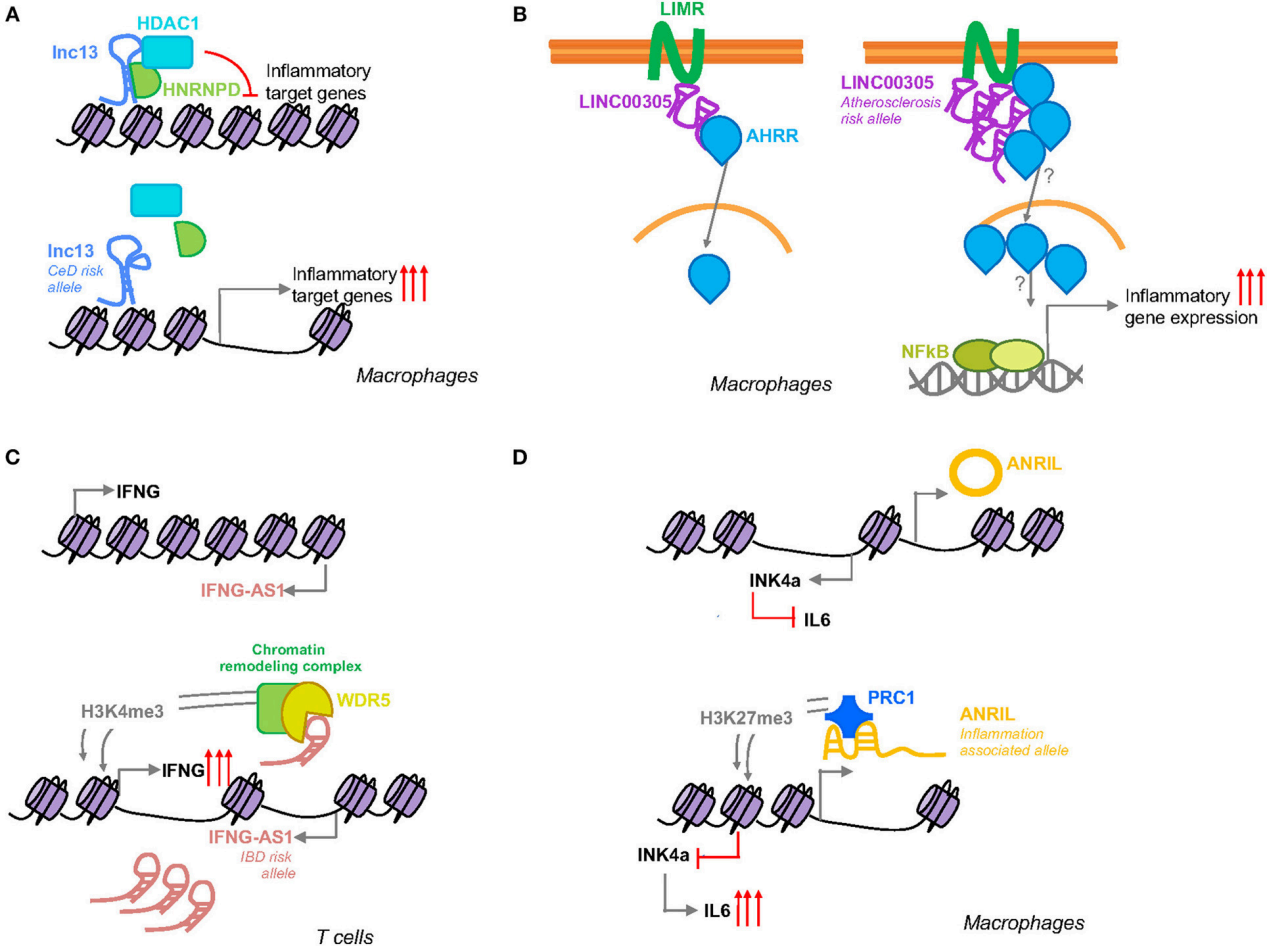
Schematic representation of the function of inflammation associated SNP harboring lncRNAs. **(A)** *Lnc13* harbors a CeD associated SNP that changes the secondary structure of the lncRNA modifying its binding with the proteins hnRNPD and HDAC1 and regulating the expression of disease related inflammatory genes. **(B)** *LINC00305* interacts with the transmembrane protein LIMR facilitating the binding of this protein to AHRR that in turn induces the translocation of the latter to the nucleus inducing NFκB and subsequent inflammatory gene expression. Atherosclerosis patients present higher levels of this lincRNA which could be influenced by a SNP located in the promoter region of *LINC00305*. **(C)** *IFNG-AS1* is closely located to *IFNG* gene. Activation of its transcription leads to induction of *IFNG* by WDR5 mediated H3K4me3 methylation. IBD patients present higher levels of *IFNG-AS1* that could be related to a disease associated SNP located in the enhancer region of the lncRNA. **(D)** Suggested SNP related splicing model for *ANRIL* mediated inflammation regulation. The inflammation associated allele will affect *ANRIL* splicing generating a linear *ANRIL* that will interact with a member of the PRC1 complex mediating an epigenetic transcriptional repression of the *INK4a* gene via H3K27me3.

This lncRNA is expressed in different human cells and tissues, including mononuclear cells in the lamina propria, where it was observed to be localized in the nucleus. *Lnc13* quantification in small intestinal biopsy samples from celiac patients and controls showed markedly lower levels of this lncRNA in CeD samples, contrary to the expression of the coding mRNA, *IL18RAP* (42). In fact, it is known that *IL18RAP* expression is induced in response to inflammation via NF-κB in certain immune cells (43). The characterization of the regulation, function and mechanisms of action of *lnc13* revealed that under basal conditions *lnc13* represses the expression of certain CeD related genes (*STAT1, MYD88, IL1RA*, and *TRAF2*) via its interaction with hnRNPD (Heterogeneous Nuclear Ribonucleoprotein D), a nuclear AU1 rich RNA binding protein, and HDAC1 (Histone Deacetylase 1), a histone deacetylase which negatively regulates transcription, proteins on the chromatin (Figure 2A). In response to inflammatory stimuli, *lnc13* is degraded by Decapping enzyme 2 (DCP2), releasing the protein complex from chromatin and allowing the expression of the proinflammatory genes (37).

Although the GWAS disease association has been generally attributed to the SNP rs917997 (33), located 1.5 kb away from the coding gene, linkage analysis of the *lnc13* region revealed that there are a total of six SNPs in total linkage disequilibrium within the lncRNA sequence. The nucleotide changes in *lnc13* cause a disruption of the secondary structure of this lncRNA decreasing its affinity to bind hnRNPD and chromatin, resulting in higher expression of the proinflammatory genes (37). Thus, patients with the risk haplotype have a higher basal expression of CeD related inflammatory genes, thereby increasing their predisposition to develop the disease (Figure 2A).

The SNPs in *lnc13* have also been associated with other inflammatory diseases, such as T1D, Crohn's disease or rheumatoid arthritis (44–46). Interestingly, while the risk signal in CeD corresponds to the T allele, the C allele is the risk allele in T1D, suggesting that the function of the lncRNA may be cell specific, but equally affected by the SNPs in different inflammatory diseases.

In summary, it is known that *lnc13* and the CeD associated SNP rs917997 contribute to the pathology of celiac disease by regulating expression of certain immune related genes that play a role in the development of inflammation in the intestinal epithelia. However, other cell-type and allele specific functions cannot be excluded, due to the association of this SNP with other inflammatory conditions.

## *LINC00305* and Inflammatory Response in Atherosclerosis

Atherosclerosis is a complex, chronic disease of the arterial wall triggered by multiple factors including amongst others, inflammation and lipid metabolism (47). Monocyte-mediated inflammation plays a critical role in atherosclerosis due to the secretion of proinflammatory cytokines and amplification of local inflammation (48).

Although a locus in the 9p21.3 region is the strongest genetic factor for atherosclerosis described so far, GWAS have led to the identification of a substantial number of additional genetic loci associated with atherosclerosis and atherosclerosis-related complications (49). Analysis of atherosclerosis GWAS SNPs, revealed that the SNP rs2850711 is located within an intronic sequence of a long intergenic non-coding RNA named *LINC00305*. This lincRNA was found to be overexpressed in atherosclerotic plaques and in peripheral blood mononuclear cells (PBMCs) from patients, supporting its role in the disease. Analysis of *LINC00305* in the cell types that composed the plaques revealed that monocytes are the primary cell type expressing this lncRNA and that its expression was induced in response to stimulation with lipopolysaccharide (LPS). Further functional studies, demonstrated that *LINC00305* interacts with the transmembrane protein LIMR (Lipocalin-1 Interacting Membrane Receptor) enhancing its interaction with AHRR (Aryl-Hydrocarbon Receptor Repressor) which at the same time promotes NF-κB activation and subsequent inflammatory gene expression (Figure 2B) (50).

The development of atherosclerotic plaques is induced by the change in phenotype of the vascular smooth muscle cells in response to the cytokines secreted by inflammatory cells (51). To investigate the functional significance of *LINC00305* in the pathogenesis of atherosclerosis, the lincRNA was stably overexpressed in human monocytes and these were co-cultured with human aortic smooth muscle cells. It was observed that those muscle cells that were cultured in the presence of the monocytes overexpressing *LINC00305* showed less expression of their basal markers, thus suggesting that they were switching to the pathogenic phenotype (50). Independent studies, have shown that overexpression of this lincRNA induces apoptosis in hypoxia induced endothelial cells (52). Further analysis of the role of this lincRNA in the regulation of apoptosis, revealed that it acts as an endogenous sponge for miR-136 which had been previously related to apoptosis in the context of atherosclerosis (52, 53).

Although it is quite clear that *LINC00305* plays a functional role in development of atherosclerosis by inducing production of inflammatory cytokines in monocytes, and by regulating apoptosis via miR-136, the role of the associated SNP in the function of the lincRNA remains to be elucidated. The GWAS SNP rs2850711 is transmitted in a linkage disequilibrium (LD) block of a total of 16 SNPs, all of which are located within introns, and hence probably do not influence the secondary structure of the lincRNA. Although there are no *in vitro* molecular studies evaluating this possibility, it is noteworthy that one of the associated SNPs lies within an experimentally confirmed USF2 (Upstream Transcription Factor 2) binding region (54). As USF2 is a protein that has been associated with cholesterol metabolism and atherosclerosis development (55), further mechanistic studies assessing the contribution of the SNP alleles in the function and regulation of the lincRNA are necessary to understand how the SNPs in non-coding regions identified by GWAS influence the inflammatory environment in atherosclerosis.

## *IFNG-AS1* (NeST or Tmevpg1) and Ulcerative Colitis

Inflammatory bowel diseases (IBD) are chronic common inflammatory gastrointestinal disorders clinically comprised of Crohn's disease (CD) and ulcerative colitis (UC) (56). These diseases are believed to develop due to inappropriate inflammatory responses to intestinal microbes and foreign antigens in genetically susceptible individuals (57, 58).

Meta-analyses of multiple GWAS have implicated 163 genetic loci in IBD susceptibility. Although functional analysis of the associated SNPs have revealed multiple pathophysiological mechanisms, the function for many of the genes in close association with these loci are yet to be determined (59, 60). It has been observed that several lncRNAs are differentially expressed in inflammatory bowel disease, and that the expression profiling of lncRNAs can be useful to stratify IBD patients from healthy controls (27, 61, 62).

When comparing the genomic location of differentially expressed lncRNAs with those of IBD susceptibility loci, *IFNG-AS1* (also called *NeST* or *Tmevpg1*) was found to fulfill both criteria (61). The IBD associated SNP rs7134599 is located in the region 12q15 in close proximity to the inflammatory cytokine *IFNG*. This SNP is in total LD with 10 other SNPs within the lncRNA gene. Additionally, *IFNG-AS1* is significantly overexpressed in intestinal samples of ulcerative colitis patients and its expression appears to correlate with the elevated levels of *IFNG, IL1, IL6*, and *TNF-*α observed in patients (61). Increased expression level of this lncRNA has also been related to other inflammatory diseases, such as Hashimoto's thyroiditis or Sjögren syndrome (63, 64) although the mechanisms by which it contributes to development of these diseases remain unclear.

*IFNG-AS1* gene was first related with the immune response in the context of susceptibility to persistent Theiler's virus infection. It was observed that *IFNG-AS1* is expressed in immune cells of mouse and human origin and it was speculated that this lncRNA may regulate the expression of *IFNG* (65). Further studies, demonstrated that *IFNG-AS1* contributes to *IFNG* expression regulation as part of the Th1 differentiation program and that T-bet guides epigenetic remodeling of the lncRNA enhancers, leading to recruitment of stimulus-inducible transcription factors, such as NF-κB (66, 67). More recently, it was observed that different mouse strains with different genotype composition of *IFNG-AS1* were sufficient to confer disparate immune-related phenotypes. Specifically, certain alleles, derived from SJL/J mouse strain, were responsible for the failure to clear Theiler's virus, but at the same time they conferred resistance to lethal infection with *Salmonella enterica Typhimurium* and induced synthesis of *Ifng* in CD8^+^ T cells. Functional analysis performed in this same study, showed that *IFNG-AS1* is a nuclear lncRNA that can act in trans. *IFNG-AS1* binds WDR5, a component of active chromatin remodeling complexes increasing H3K4me3 methylation which in turn programs an active chromatin state that induces *Ifng* gene transcription (68) (Figure 2C).

Although *IFNG-AS1* is differentially expressed in IBD samples and harbors a disease associated SNP (61) the exact impact of the different alleles in the development of the disease has not been assessed so far. *In silico* interaction evaluation of human *IFNG-AS1* lncRNA and WDR5 protein using CatRapid (69), an algorithm that estimates the binding propensity of protein-RNA pairs, states that these two molecules are also able to interact, suggesting that it may act in a similar way as described in mice. Analysis of the location of the SNPs that are in LD with the associated SNP rs7134599 reveals that all of them are located in intronic regions, suggesting that they will most likely not affect the secondary structure of the RNA molecule. However, analysis of the region using HaploReg v4.1 (70) shows that 4 of the SNPs are located within enhancer histone marks and all of them are predicted to disturb a protein binding motif that could change the regulation of the lncRNA expression, thereby influencing the levels of *IFNG*.

Thus, *INFG-AS1* is clearly involved in the immune response and inflammatory processes involved in disease, and although *in silico* data point to a disturbance of lncRNA expression regulation mediated by the IBD associated SNPs uncovered by GWAS, the true relevance of these SNPs have still to be experimentally confirmed.

## *ANRIL* and Inflammation

The antisense non-coding RNA in the INK4 locus or *ANRIL* was first described in melanoma patients (71) and since its discovery it has been shown to be involved in several types of cancers (72). This lncRNA is located in the 9p21 region, that has been associated by GWAS not only to cancer but also to other diseases that are related with inflammation, such as coronary artery disease (73) or type 2 diabetes (T2D) (74). *ANRIL* is expressed as either linear or circular forms, that have been observed to have opposing effects in disease development (75), making the deciphering of the functionality of this lncRNA and the involvement of its related SNPs highly complicated.

*ANRIL* has been described to interact with *CBX7* (Chromobox 7), one of the members of the polycomb repressive complex 1 (PRC1). *CBX7* binds both, *ANRIL* and H3K27me3 to mediate an epigenetic transcriptional repression of the *INK4a* (Inhibitor of CDK4) gene, which is located adjacent to the *ANRIL* gene (Figure 2D) (76). INK4a is a cell cycle inhibitor that is lost in a wide spectrum of cancers (77), but it has been also been reported to act as an anti-inflammatory molecule that is able to suppress the production of IL-6 in macrophages (78). *ANRIL* itself has been also shown to regulate the inflammatory response by its interaction with the YY1 (Yin Yang 1) protein (79), a transcription repressor involved in cancer development and immune processes (80).

The influence on gene expression of the variants within *ANRIL* region have been analyzed in a variety of tissues and cells, but the results have been inconsistent (75). Several SNPs have been described to be involved in alternative splicing events, and modifying *ANRIL* structure has been suggested to lead to changes in its function and consequent regulation of downstream inflammatory genes (Figure 2D) (22). Although the exact mechanism by which the SNPs within *ANRIL* confer susceptibility to disease has not been firmly established, it seems clear that the disease associated SNPs are related with *ANRIL* expression, suggesting that modulation of its expression mediates disease susceptibility.

One example of SNP-dependent *ANRIL* related inflammation is the correlation between the lead periodontitis associated SNP, rs1333048, and the levels of the C-reactive protein. Periodontitis is a complex, chronic inflammatory disease associated with increased concentration of high-sensitive C-reactive protein (hsCRP), a marker for systemic inflammation. It was found that AA-genotype of *ANRIL* rs1333048 is associated with significantly elevated hsCRP plasma levels in patients with periodontitis (81). However, the functional relationship between the SNP, *ANRIL* and the hsCRP molecule has not been clearly established.

Another disease in which *ANRIL* has been functionally implicated is Type 2 diabetes (T2D). Although the major causes of T2D are insulin resistance and beta cell dysfunction, recent evidence implicates the immune system in the pathogenesis of this disease that can be considered as an autoinflammatory disease (82). T2D associated SNPs in the *ANRIL* locus were evaluated, and it was observed that the risk genotype was correlated with increased levels of *ANRIL* expression. Moreover, although the associated SNPs did not seem to influence insulin secretion, it was observed that they affect human beta cell proliferation index, with homozygous risk alleles showing approximately half of the proliferation capacity observed in the presence of the protective alleles (83). Although this study suggests that ANRIL lncRNA may play a role in human islets and uncovers a link between T2D associated SNPs and beta cell proliferation, once again, the functional relationship between the SNPs and the biological process is still not understood.

Additionally, *ANRIL* is significantly downregulated in the inflamed intestinal mucosa of Crohn's and inflammatory bowel disease patients (84). At the same time its reduced levels in rats have been related to prevention of coronary atherosclerosis due to lower expression of inflammatory factors (85) which are upregulated in patients with coronary artery disease (86).

It therefore seems clear that disease associated SNPs in *ANRIL* lncRNA influence its function in the context of inflammatory diseases. However, the involvement of *ANRIL* in inflammation and the influence of the GWAS SNPs in the function of the different isoforms of *ANRIL* needs further investigation.

## Concluding Remarks

Although our knowledge about the genetic variants contributing to immune mediated diseases has increased considerably in the last decade, the intergenic location of the great majority of the associated SNPs has made it difficult to decipher their functional roles in disease development. As disease associated SNPs are enriched within lncRNAs, and as many of these RNA molecules have been implicated in the regulation of inflammatory processes, a new field of study focused on the influence of disease-associated SNPs in the function of inflammation-related lncRNAs has been opened. Interestingly, such lncRNAs have been linked to major immune-mediated diseases as celiac disease, type 1 diabetes or rheumatoid arthritis. The experimental approaches utilized so far have been mainly focused on the expression analysis of the SNP harboring lncRNA in diseased tissues, but functional studies evaluating the contribution of each allele to lncRNA function, and thus to disease development, is mostly missing. In general, the function of the lncRNA itself, and the mechanisms by which they contribute to inflammatory disease development, are mostly uncharacterized. Analyzing the position and the linkage disequilibrium block of the associated SNP within the lncRNA sequence can help predict the functional impact of the allelic variant. Associated SNPs may not only affect the expression of the lncRNA itself, but also their splicing, their secondary structure or their ability to regulate expression of downstream genes. Thus, approaches that evaluate the functional differences of the lncRNA alleles are necessary in order to understand how the disease-associated SNPs affect the function of such inflammation related lncRNAs.

As our knowledge about the molecular mechanisms by which the inflammation related lncRNAs exert their biological functions increases, so will our understanding of how the disease associated SNPs influence lncRNA function thereby opening up the possibility for targeting such lncRNAs for diagnostic and therapeutic purposes.

## Author Contributions

All authors listed have made a substantial, direct and intellectual contribution to the work, and approved it for publication.

### Conflict of Interest Statement

The authors declare that the research was conducted in the absence of any commercial or financial relationships that could be construed as a potential conflict of interest.
